# Supplementary material for: Impact of CFTR Modulators on Beta-Cell Function in Children and Young Adults with Cystic Fibrosis
Source: J Clin Med. 2022 Jul 17;11(14):4149. doi: 10.3390/jcm11144149 (PMC9319690; doi:10.3390/jcm11144149)
Supplement: Supplementary file 1 [file jcm-11-04149-s001.zip › jcm-1751349-supplementary.pdf]

Supplementary Table S1. Additional metabolic characteristics of the study subjects before starting Lumacaftor/Ivacaftor and after 12-18 months of treatment. Data are expressed as mean (standard deviation) or median (IQR), unless otherwise specified. Abbreviations: *HbA1c* glycated haemoglobin; *PG* plasma glucose; *min* minutes; *PI* plasma insulin; *CP* C-peptide; *IGI* Insulinogenic Index; *DI* Disposition Index; *HOMA-IR* Homeostasis model assessment insulin resistance.

**Variables**	**Baseline**	**After 12-18 months of Lumacaftor/Ivacaftor**	***p***
HbA1c (%, mmol/mol)	5.8 (5.3 – 6.1), 39.5 (37.2 – 42.5)	5.6 (5.4 – 5.9), 38.0 (36.2 – 40.5)	0.59
Fasting PG (mg/dL, mmol/L)	81.5 [77.5 – 87.8], 4.52 [4.29 – 4.86]	89.0 [82.5 – 94.8], 4.94 [4.57 – 5.25]	**0.032**
30 min PG (mg/dL, mmol/L)	132.0 [123.0 - 157.8], 7.32 [6-82 – 8.75]	146.0 [132.6 – 154.0], 8.10 [7.37 - 8.55]	0.71
60 min PG (mg/dL, mmol/L)	159.0 [133.3-187.8], 8.82 [7.39 – 10.42]	183.0 [156.8 – 207.8], 10.15 [8.70 – 11.53]	0.45
90 min PG (mg/dL, mmol/L)	138.0 [119.0 -196.0], 7.66 [6.60 - 10.88]	149.0 [131.8 – 165.5], 8.27 [7.31 – 9.18]	0.87
120 min PG (mg/dL, mmol/L)	121.0 [105.0 - 157.5], 6.71 [5.83 – 8.74]	125.0 [108.3 – 143.3], 6.94 [6.05 – 7.95]	0.69
Fasting PI (pmol/L)	37.2 [24.3 - 75.2]	49.0 [29.0 – 58.0]	0.87
30 min PI(pmol/L)	132.3 [97.7 – 249.8]	169.5 [140.8 - 266.9]	0.68
60 min PI (pmol/L)	258.0 [193.8 – 393.4]	315.0 [210.8 – 513.4]	0.57
90 min PI (pmol/L)	379.2 [189.0 – 472.4]	391.7 [273.1 - 500.6]	0.50
120 min PI(pmol/L)	263.9 [126.4 – 533.0]	274.3 [224.7 – 412.9]	0.75
Fasting CP (nmol/L)	0.37 [0.21 – 0.59]	0.37 [0.31 – 0.50]	0.97
30 min CP (nmol/L)	1.11 [0.54 – 2.08]	1.08 [0.83 – 1.30]	0.82
60 min CP (nmol/L)	1.78[1.12 - 3.01]	2.05 [1.39 – 2.47]	0.97
90 min CP (nmol/L)	2.25 [1.70 – 2.99]	2.29 [1.97 – 2.87]	0.24
120 min CP (nmol/L)	2.18 [1.30 - 3.64]	2.38 [1.80 – 3.02]	0.68
IGI	0.29 [0.21 – 0.42]	0.31 [0.24 – 0.56]	0.75
DI	2.40 [1.78 – 3.16]	1.88 [1.41 – 3.17]	0.60
HOMA-IR	1.07 [0.68 – 2.00]	1.43 [0.98 – 1.99]	0.60
Matsuda Index	7.88 [4.20 – 11.02]	5.46 [3.98 – 6.98]	0.25

Supplementary Table S2. Additional metabolic characteristics of the study subjects before starting Exacaftor/Ivacaftor/Tezacaftor therapy and after 12-18 months of treatment. Data are expressed as mean (standard deviation) or median [IQR], unless otherwise specified.Abbreviations: *HbA1c* glycated haemoglobin; *PG* plasma glucose; *min* minutes; *PI* plasma insulin; *CP* C-peptide; *IGI* Insulinogenic Index; *DI* Disposition Index; *HOMA-IR* Homeostasis model assessment insulin resistance.

**Variables**	**Baseline**	**After 12-18 months of Exacaftor/Ivacaftor/Tezacaftor**	**p**
HbA1c (%, mmol/mol)	5.8 [5.2-6.6], 40.0 [33.0-48.5]	5.4 [4.8 – 5.8], 36.0 [29.5-40.0]	**0.04**
Fasting PG (mg/dL, mmol/L)	84.0 [71.50 – 104.0], 4.66 [3.97-5.77]	90.0 [86.5-103], 4.99 [4.80-5.72]	0.50
30 min PG (mg/dL, mmol/L)	161.0 [130.0-217.5], 8.94 [7.22-12.07]	180.00 [146.0-209.0], 9.99 [8.10-11.60]	0.59
60 min PG (mg/dL, mmol/L)	221.0 [129.0-261.5], 12.27 [7.16-14.52]	183.0 [149.5-246.0], 10.16 [8.30-13.66]	0.69
90 min PG (mg/dL, mmol/L)	181.0 [104.0-238.5], 10.05 [5.77-13.24]	189.0 [130.0-227.5], 10.49 [7.22-16.62]	0.69
120 min PG (mg/dL, mmol/L)	133.00 [103.0-202.0], 7.38 [5.72-11.21]	142.00 [111.0-182.5], 7.88 [6.16-10.13]	0.89
Fasting PI (pmol/L)	34.73 [16.67-75.35]	46.53 [24.31-75.35]	0.69
30 min PI (pmol/L)	214.6 [111.5-339.6]	275.0 [174.3-284.4]	0.69
60 min PI (pmol/L)	323.6 [171.5-704.6]	323.6 [214.6-677.1]	1.00
90 min PI (pmol/L)	316.7 [181.3-509.8]	397.3 [284.8-771.2]	0.23
120 min PI (pmol/L)	310.4 [236.1-443.1]	425.7 [238.2-594.8]	0.14
Fasting CP (nmol/L)	0.34 [0.30-0.54]	0.49 [0.42-0.55]	0.35
30 min CP (nmol/L)	1.12 [0.65-1.50]	1.26 [0.86-1.81]	0.08
60 min CP (nmol/L)	2.00 [1.07-3.26]	2.30 [1.46-2.89]	0.69
90 min CP (nmol/L)	1.67 [1.56-3.19]	2.42 [1.76-3.85]	0.23
120 min CP (nmol/L)	1.92 [1.67-3.13]	2.85 [2.06-3.12]	0.5
IGI	0.25 [0.13-0.62]	0.33 [0.31-0.34]	0.15
DI	1.22 [0.36-4.48]	2.64 [1.05-4.49]	0.72
HOMA-IR	2.10 [0.45-5.40]	1.40 [0.75-2.70]	0.50
Matsuda Index	4.88 [3.54-7.95]	8.14 [3.15-14.51]	0.47
